# Geographic isolation facilitates the evolution of reproductive isolation and morphological divergence

**DOI:** 10.1002/ece3.3474

**Published:** 2017-10-27

**Authors:** McLean L. D. Worsham, Eric P. Julius, Chris C. Nice, Peter H. Diaz, David G. Huffman

**Affiliations:** ^1^ Department of Biology Texas State University San Marcos TX USA; ^2^ Department of Zoology University of Hawaii Honolulu HI USA; ^3^ U.S. Fish and Wildlife Service Texas Fish and Wildlife Conservation Office San Marcos TX USA

**Keywords:** evolution, geographic isolation, molecular diversity, morphological diversity, reproductive isolation

## Abstract

Geographic isolation is known to contribute to divergent evolution, resulting in unique phenotypes. Oftentimes morphologically distinct populations are found to be interfertile while reproductive isolation is found to exist within nominal morphological species revealing the existence of cryptic species. These disparities can be difficult to predict or explain especially when they do not reflect an inferred history of common ancestry which suggests that environmental factors affect the nature of ecological divergence. A series of laboratory experiments and observational studies were used to address what role biogeographic factors may play in the ecological divergence of *Hyalella* amphipods. It was found that geographic isolation plays a key role in the evolution of reproductive isolation and divergent morphology and that divergence cannot be explained by molecular genetic variation.

## 1 INTRODUCTION

Geographic isolation in novel environments often results in rapid (Eroukhmanoff, Hargeby, & Svensson, 2009) parallel and convergent evolution (Eroukhmanoff et al., 2009; Muschick, Indermaur, & Salzburger, 2012). Reproductive isolation has been shown to evolve rapidly in populations adapting to novel environments (Hendry, Wenburg, Bentzen, Volk, & Quinn, 2000), presumably resulting in ecological speciation. However, identifying and quantifying the potentially multifarious processes that contribute to the evolution of reproductive isolation remains a challenge (Garant, Forde, & Hendry, 2007; Nosil, Harmon, & Seehausen, 2009; Nosil et al., 2012; Rundell & Price, 2009). These processes might include ecological, physiological, or morphological adaptation to novel environments, along with biogeographic processes that promote differentiation or limit gene flow. Identifying the contributors to reproductive isolation can be especially difficult in recently diverged, or rapidly diverging lineages, or in lineages that contain cryptic diversity. For example, morphological similarity is not a reliable predictor of interfertility among cryptic lineages. Reproductive isolation has been found to exist within nominal morphological species revealing the existence of cryptic species complexes (Dincă et al., 2013; Gebiola, Kelly, Hammerstein, Giorgini, & Hunter, 2016; Ishikawa et al., 2013; Paterson et al., 2016). Cryptic species complexes may also be paraphyletic in many instances due to selection driving morphological conformity across several unrelated populations occurring in similar habitats (Butlin et al., 2014; Westram, Panova, Galindo, & Butlin, 2016). Despite these challenges, cases of cryptic divergence provide opportunities for study of the evolution of reproductive isolation (Rosenblum & Harmon, 2011) and the development of approaches that can be used to test hypotheses about the factors contributing to reproductive isolation.

Herein, we combine data on morphological and molecular genetic variation with experimental quantification of the strength of reproductive isolation among populations with varying degrees of geographic isolation. We focused on the freshwater genus of talitrid amphipods, *Hyalella* (Smith 1874). The nominal species *H. azteca* (Gonzalez & Watling, 2002) has been found to contain extensive cryptic diversity (Dionne, Dufresne, & Nozais, 2017; Dionne, Vergilino, Dufresne, Charles, & Nozais, 2011; Vergilino, Dionne, Nozais, Dufresne, & Belzile, 2012; Witt & Hebert, 2000; Witt, Threloff, & Hebert, 2006). Also belonging to this genus are numerous morphologically distinct nominal species, each endemic to just a single locality (Baldinger, 2004; Baldinger, Shepard, & Threloff, 2000; Cole & Watkins, 1977; Stevenson & Peden, 1973; Witt et al., 2006). Some of these populations have been found to occur sympatrically with populations of *H. *cf*. azteca* (Cole & Watkins, 1977; Stevenson & Peden, 1973; Witt et al., 2006), suggesting that reproductive isolation has allowed the two forms to coexist sympatrically without the endemic form going extinct due to introgression. This assertion is supported by al lack of evidence for niche partitioning among sympatrically occurring populations of *Hyalella* (Dionne et al., 2017). The presence of cryptic lineages, variation in the degree of geographic isolation among lineages, and the evidence of local adaptation in the narrowly distributed lineages makes *Hyalella* an ideal system for quantifying the factors that contribute to the evolution of reproductive isolation.

The objectives of this study were to address three questions regarding the patterns of reproductive isolation among *Hyalella* lineages: (i) “What role does geographic isolation play in determining the patterns of molecular and morphological differentiation?”, (ii) “How are levels of reproductive isolation related to morphological and molecular genetic differentiation?”, and (iii) “Can variation in reproductive isolation be explained by biogeography?” In order to answer these questions, we surveyed morphological and molecular genetic variation, and experimentally quantified the strength of reproductive isolation among lineages, and the answers to these questions contribute to a foundation for understanding the multifarious processes that shape the evolution of reproductive isolation.

## 2 METHODS

This study used five populations (Table 1) belonging to *Hyalella*—a widespread and abundant taxon of freshwater amphipods distributed across North America. Two of the populations studied herein fit the expectations of the *H. *cf. *azteca* morphotype (as defined by Gonzalez & Watling, 2002) while the other three are noticeably morphologically distinct. However, only one of these populations has been formally described (*Hyalella texana* Stevenson & Peden, 1973). One of our sampling locations (San Marcos Springs; referred to herein as SMS) was found to have a population of *H. *cf. *azteca* co‐occurring with a morphologically distinct undescribed spring‐endemic species (referred to herein as SMS *Hyalella* sp.). SMS *Hyalella* sp. and *H. texana* are both documented to be endemic to physicochemically stable springs separated by hundreds of kilometers of ambient surface water, suggesting that physiological limitations are responsible for the geographic isolation of these populations. We compared morphological and genetic variation, in combination with attempted mating experiments and study of biogeographic distributions, in an attempt to explain factors contributing to reproductive isolation.

**Table 1:** Collection localities and count of dorsal mucronation for each *Hyalella* population

Collection locality	Coordinates	Population	Modal dorsal mucronation count (*n*, range)
Devils River	29°53′58.45″N, 100°59′51.17″W	Devils *Hyalella* sp. (widespread)	2 (20, 0–2)
Comal River	29°42′38.00″N, 98°7′39.60″W	*H. *cf. *azteca* (widespread)	2 (20, 2–2)
San Marcos River	29°53′27.42°N”, 97°55′56.73″W	*H. *cf. *azteca* (widespread)	2 (20, 2–2)
San Marcos River	29°53′36.10″N, 97°55′52.80″W	SMS *Hyalella* sp. (spring endemic)	3 (20, 3–4)
Clear Creek Springs	30°54′22.20″N, 99°57′29.20″W	*H. texana* (spring endemic)	4 (20, 3–4)

### 2.1 Establishment of stock cultures

Stock cultures of *Hyalella* were established to provide a continuous source of live animals for experimentation and to control for the possibility of morphological differentiation due to phenotypic plasticity in situ. Cultures of *Hyalella* spp. were collected from the four localities listed in Table 1. All amphipods were collected from source localities using dip nets, turkey basters, or a Ponar grab sampler. Cultures were established with at least three separate sampling events for each population between January and August of 2014 and maintained under essentially identical conditions in separate 20‐L buckets for each population. Each bucket was given a sand substrate and filled with artesian water with water changes twice monthly. Buckets were maintained at a constant 22°C and kept on a 12 hr/12 hr‐light/dark cycle. All cultures were fed the same diet of *Amblystegium* sp. and organic detritus ad libitum on a daily basis. Great care was taken when handling cultures to ensure that organisms did not get moved between cultures.

### 2.2 Quantifying morphological variation

To quantify morphological variation while controlling for potential effects of phenotypic plasticity, cultures from all five populations were reared in common‐garden replicates. As conditions in all stock cultures (described above) were maintained under the same conditions, we anticipated that five generations would be sufficient to control for environmental or maternal effects; a lack of variation in neonate size across all the populations and generations in captivity suggests that maternal effect was not a factor (Glazier, 2000; Table S3). Therefore, after at least five generations of raising amphipods in stock cultures, morphology was compared between cultures. Twenty individuals were gently wet‐mounted (taking care to avoid harming experimental individuals) and photographed at 10× magnification with a calibrated scale bar superimposed on each photograph using an Olympus cellSens camera system and software. Morphometric characters (total length, longest mucronation length, and head length; see Figure S1 for explanation) were estimated from these photographs using Digimizer software (www.digimizer.com). We also counted the number of dorsal mucronations and calculated the ratio of the length of the longest mucronation to total length.

Principal components analysis (PCA) was used to examine morphological characteristics of wild‐caught populations. The PCA analysis included three independent variables: head length/total length ratio and longest spine as continuous variables, and spine count as a meristic variable. PCA was conducted in R using the “princomp” function.

Using the same morphological variables, the degree that common‐garden populations morphologically differentiated from wild‐caught populations, if at all, was assessed. Pairwise permutational multivariate analysis of variance (perMANOVA) was used to test for differences across wild‐caught populations, across cultured populations, and between wild‐caught and cultured populations using Bray–Curtis distance and the “Adonis” function using the statistical package “vegan” in R. For each perMANOVA analysis, a sequential Bonferroni was applied to the results. The statistical package vegan in R was used for the perMANOVA analyses (Oksanen et al., 2016).

To test for a relationship between morphological and molecular variation among *Hyalella*, the Euclidean distance between the centroids of each population in PCA space was compared to pairwise Bayesian model‐corrected genetic distances using the “ade4” package (Dray & Dufour, 2007) in R. The relationship between morphology and phylogeny was visualized using the R package “phytools” (Revell, 2012).

### 2.3 Molecular methods

A molecular phylogeny based on the mitochondrial cytochrome C oxidase subunit I (COI) locus was constructed in order to analyze the relationship between morphological similarity, geographic factors, and a history of shared common ancestry. During collection of organisms for the establishment of stock cultures, some specimens were preserved in 95% EtOH and stored at room temperature. DNA was extracted from these individuals (*n *=* *3 per population) by placing all or part of individuals in microtubes containing a chelating resin (Chelex 100, Sigma Aldrich), heated to 60°C for 20 min, then 100°C for 20 min. A fragment of the COI gene was PCR‐amplified using the primer TrpPar1 (5′—GTTATATAAACTATTAGCCTTCCAA—3′) paired with either COIaV9 (5′—ACTGCCACAACAGAYAARTAMGACCC—3′) or COIaV10 (5′—ACAGCAACAACAGATAARTARGACC—3′). PCR was carried out with TopTaq (Qiagen) kits in 50‐μl reactions containing 2.0 μl of template DNA. Cycling conditions were 5 min at 94°C, followed by 35 cycles of 45 s at 94°C, 45s at 51°C, and 60 s at 72°C, followed by 5 min at 72°C. PCR products were gel purified and sequenced with TrpPar1 (COI) using an ABI 3730 automated sequencer.

All sequences generated by this study were queried in GenBank using a BLAST search (blast.ncbi.nlm.nih.gov) which returned 332 *Hyalella* sequences (including the 15 sequences generated herein) as well as 242 sequences belonging to ten other families of amphipods (a subset of one sequence per amphipod family was randomly selected to serve as outgroups). Of these 332 *Hyalella* sequences, geographic data were available for 269; therefore, only these 269 sequences were retained for further analysis. Additional sequences belonging to amphipods in the families Chiltoniidae, Gammaridae, Gammarellidae, Ischyroceridae, Lysianassidae, Metacrangonyctidae, Niphargidae, and Talitridae were compiled into an alignment with *Hyalella* sequences to serve as outgroups and to provide context for the depth of divergence within *Hyalella*. The resulting alignment was trimmed to 500‐bp to remove missing data using Geneious R9 (Kearse et al., 2012). After trimming the alignment, a matrix of pairwise comparisons of genetic dissimilarity including all 269 *Hyalella* sequences was constructed using MEGA7 (Kumar, Stecher, & Tamura, 2016). This pairwise matrix was used to infer the geographic distributions of each haplotype of *Hyalella*, as well as to remove redundant sequences of each haplotype before further analysis (sequences with 0.000 pairwise divergence were considered the same haplotype).

Because the COI locus is protein coding, a consensus sequence was computed for all *Hyalella* sequences using Geneious and was then translated to infer the open reading frame using ORF Finder (NCBI, http://www.ncbi.nlm.nih.gov/orffinder/) to annotate codon positions. PARTITIONFINDER (Lanfear, Calcott, Ho, & Guindon, 2012) was used to select the best model of evolution for each codon position; model selection was based on the Bayesian information criterion. The best model of evolution for first and second codon positions was TVM + I + G while TVM + G was the best model for third codon positions. Phylogenies were estimated using MrBayes (Ronquist et al., 2011) with Markov chain Monte Carlo methods consisting of four Markov chains (three heated, one cold) with confidence assessed by posterior probabilities. A majority‐rule consensus phylogeny was computed by removing the first 25% of trees as burn‐in. Saturation of nucleotide substitutions was estimated by plotting uncorrected pairwise distances against the evolutionary model adjusted pairwise sequence divergence (i.e., patristic distance). Saturation was assessed by comparing the resulting slope of the regression with the theoretical slope of 1.0 of an unsaturated data set (Jeffroy, Brinkmann, Delsuc, & Philippe, 2008). Patristic distances between haplotypes were extracted from the consensus phylogeny using the “ape” library (Paradis, Claude, & Strimmer, 2004) in R. Following other authors (Major, Soucek, Giordano, Wetzel, & Soto‐Adames, 2013; Wellborn & Broughton, 2008; Witt, Blinn, & Hebert, 2003; Witt et al., 2006), taxa with 0.10 or less subst./site patristic divergence were grouped into clades to gain inference into the phylogeographic distribution of the resulting clades of *Hyalella*.

### 2.4 Quantifying reproductive isolation

To quantify reproductive isolation, we performed a series of no‐choice within (control groups) and between‐population (experimental groups) mating experiment where we attempted to achieve all possible combinations with respect to both population source and sex (Table 2). Pairings were established using stock cultures by selecting one female from one population source and selecting one male from the same (control groups) or one male from a different population (experimental groups). Only females that were not brooding eggs or young in their marsupia were selected for the experiments. Body lengths of all individuals were measured prior to pairing by gently wet‐mounting and estimating length with a calibrated reticle. Females were paired with males that were at least equal in length but not greater than twice as long in order to control for size‐assortative effects on mating success (Bollache & Cézilly, 2004). This is a conservative approach to estimating reproductive isolation because reproduction is rarely successful between pairs where males are smaller than females. Some combinations could not be achieved because it was difficult to find suitable males (i.e., males that were larger than the respective female) of the various *Hyalella* types for female *H. texana* because *H. texana* is appreciably larger in size than most other *Hyalella* species.

**Table 2:** Replication of male–female pair combinations of *Hyalella* by population source and sex

Male type	Female type				
	SMS	SMR	Comal R	Devils R	*H. texana*
SMS	**4**	1	1	1	1
SMR	2	**3**	3	2	1
Comal R	3	1	**4**	3	2
Devils R	3	1	1	**3**	3
*H. texana*	2	2	3	3	**4**

Each count represents one pair. Diagonal (bolded) is same‐population control pairs.

An experimental replicate consisted of one male and female pair. Replicates were placed individually in sealed 150‐ml containers. Each container was given the same sand substrate and fed a diet consisting of *Amblystegium* sp. and organic detritus ad libitum. Cultures were maintained at a constant 22°C, and water in containers was refreshed weekly.

Mating trials were run for 8 weeks and were checked once weekly for the production of offspring. After 8 weeks had elapsed, any pairs that had not reproduced were considered to represent unsuccessful crosses. If free swimming neonates were observed, the adults were removed. The length was measured for each of the neonates and the mean length was used to estimate age of the brood following the equation: *A = L–*1, where *A *= age in weeks and *L *= length in mm (seeTables  S3 and S4).

Estimated age was used to estimate the date that hybrid offspring had hatched and the date at which they would become 8 weeks old (since 8 weeks is the age at which most *Hyalella* species are thought to have finished most of their ontogenetic growth; Strong, 1972). At 8 weeks of age, surviving hybrid offspring were either paired with siblings or individuals other than their parents of one or both of the parental populations to test *F*_1_′s for interfertility and backcross fertility. These pairings were allowed to run for 8 weeks and were checked once weekly for the production of offspring.

### 2.5 Correlates of reproductive isolation

To evaluate potential factors that might explain the occurrence of reproductive isolation, the results from the reproductive isolation experiment were arranged into a matrix. This matrix was compared to a matrix of pairwise Bayesian model‐corrected genetic distances. Matrices were compared using the “ade4” package (Dray & Dufour, 2007) to run Mantel tests in R. To assess the possibility that the relative degree of geographic isolation may potentially lead to reproductive isolation, each population was scored as either reproductively isolated (1) or not (0). Two one‐way ANOVAs were used to test for a relationship between the reproductive isolation score and (i) the number of populations of each clade (as determined by molecular analysis), and (ii) the length of reach occupied by each population (Table 3).

**Table 3:** Factors analyzed in ANOVAs to determine if geography can account for variation in the occurrence of reproductive isolation

Population	Number of known localities	River kM	Reproductive isolation code
Comal	11	370	0
Devils	1	150	0
SMR	27	120	0
SMS	1	2	1
*Hyalella texana*	1	2	1

0 = interfertile; 1 = reproductively isolated.

## 3 RESULTS

### 3.1 Phylogenetics

**Figure 1:**
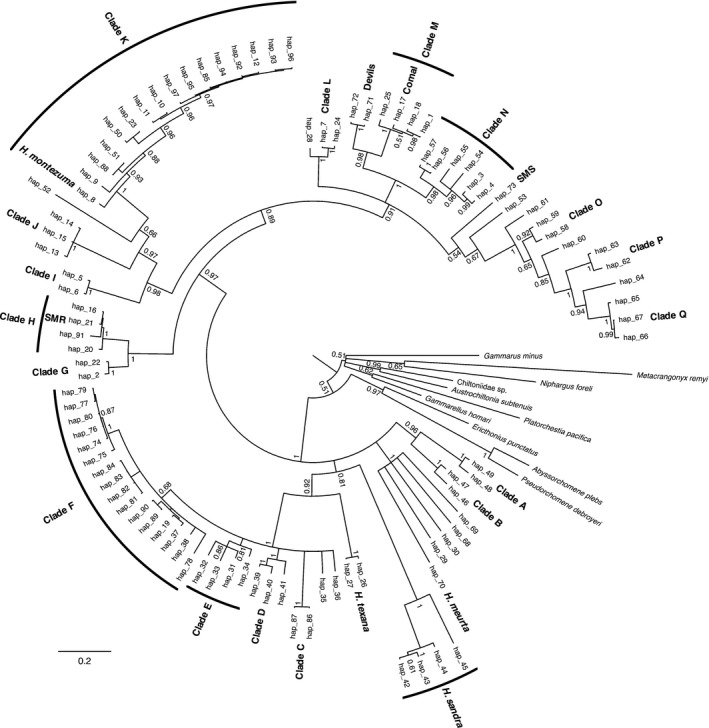
Bayesian phylogeny based on 495‐bp region of the COI gene. Terminal nodes represent unique haplotypes. Haplotypes were grouped into clades where applicable. Bayesian posterior probabilities are given at all major nodes

**Figure 2:**
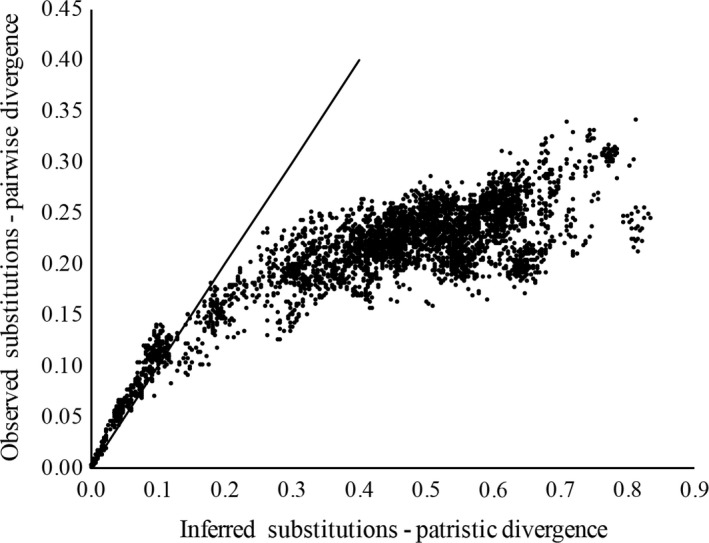
COI saturation plot. Saturation is assessed by comparing observed substitutions [pairwise uncorrected *p* distances (*Y*‐axis)] with Bayesian model‐corrected distances (*X*‐axis). The solid line has a slope of 1 and is a theoretical representation of sequence data that would occur if there was no saturation (Jeffroy et al., 2008). The observed departure from this theoretical slope (which occurs at around 0.15 substitutions per site in this data set) suggests that saturation has occurred

Pairwise comparison of 269 *Hyalella* sequences yielded 97 unique *Hyalella* haplotypes; three of the populations we sequenced had only one haplotype while the other two had two haplotypes each (Table S5). Of the 500‐bp in the alignment, 267 (53%) were variable. The protein translation was 164 amino acids spanning 495‐bp of the alignment without interruption by stop codons; therefore, subsequent analyses used the 495‐bp alignment. Using the recommended models of evolution for each codon position, the average deviation of split‐chain frequencies between runs fell to ≈0.02 after 500,000 generations and did not change by 1 million generation indicating that convergence had been reached. A majority‐rule consensus phylogeny was computed from the resulting trees (Figure 1). Appreciable molecular divergence was detected within *Hyalella* with evidence of saturation (Figure 2). To facilitate discussion of the phylogeny, haplotypes are grouped into clades (Figure 1, Table S5).

### 3.2 Evaluation of morphological variation

**Figure 3:**
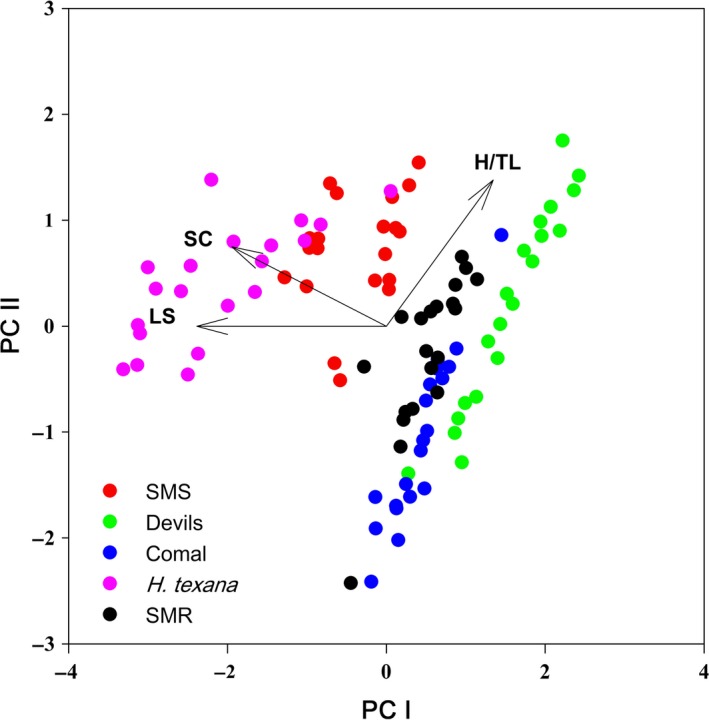
Principal components analysis plot of morphological variation of wild‐caught collections. Populations segregate with some degree of overlap. H/TL represents head length to total length ratio; SC is dorsal spine count; and LS represents length of the longest dorsal spine

**Figure 4:**
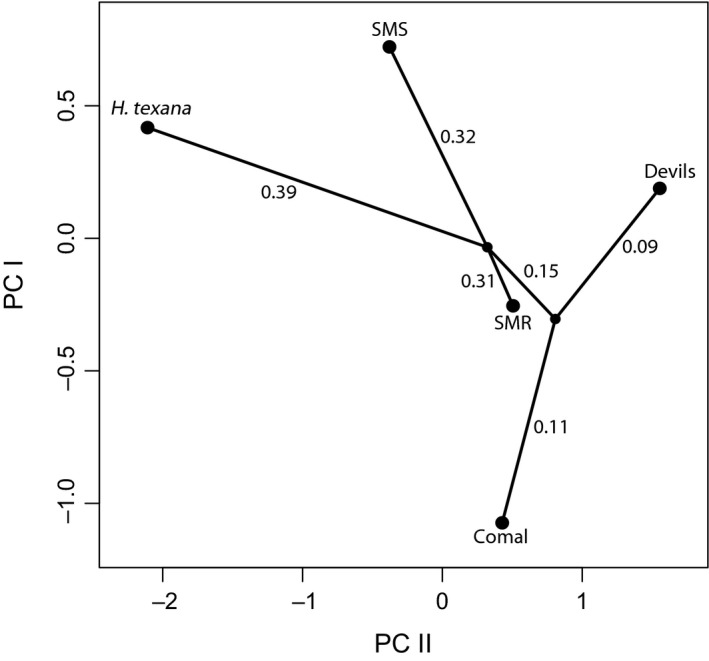
Phylomorpho plot of population centroids with phylogenetic relationship. Genetic similarity is not related to distribution of centroids in principal components analysis space. Decimals along branches represent the Bayesian model inferred number of substitutions

Principal component axes I and II cumulatively explain 89% of the morphological variation in the characters measured. PC I explained 61% of the variance while PC II explained another 28%. The morphological gradient along PC I shifted from negative loadings for longest spine and spine count to positive loadings for head/total length; PC axis II had a gradient of longest spine to head/total length (Figure 3, Table 4). PerMANOVA (as well as linear discriminant analysis and discriminant function analysis) on wild‐caught amphipods showed that all five wild‐caught amphipod populations were morphologically distinct (Table 5; Tables S1 and S2). Wild‐caught versus the common‐garden amphipods showed that all common‐garden amphipods differed significantly from their wild‐caught ancestors except for the SMS population (Table 5). Despite an apparent shift in morphology after five generations in captivity under essentially identical conditions, all common‐garden populations remained morphologically distinct from each other at *p *<* *.05 (Table 5). Distribution of centroids in PCA space was not found to be significantly correlated to genetic distance (*r *=* *0.75, *p *=* *.19; Figure 4).

**Table 4:** Variable loading from principal component analysis performed on wild‐caught collections from the five experimental amphipod populations

Variable	PC I	PC II
Longest spine	−0.680	0.000
Spine count	−0.612	0.479
Head/Total length	0.404	0.874

**Table 5:** Results of perMANOVA tests of morphometrics from wild‐caught and common‐garden stock

	*Hyalella texana*	Devils R	Comal R	SMR	SMS
*H. texana*	**0.019**	<0.001	<0.001	<0.001	<0.001
Devils R	<0.001	**<0.001**	<0.001	<0.001	<0.001
Comal R	<0.001	<0.001	**<0.001**	0.022	<0.001
SMR	<0.001	<0.001	<0.001	**0.002**	<0.001
SMS	<0.001	<0.001	<0.001	<0.001	**0.563**

All numbers are *p‐*values. The diagonal (bolded) represents the comparison of laboratory‐reared common‐garden populations with respective wild‐caught source populations. Below the diagonal are comparisons across wild‐caught populations, and above the diagonal are comparisons across common‐garden populations laboratory‐reared for five generations.

### 3.3 Reproductive isolation

After 8 weeks, all conspecific controls had successfully produced offspring while only three of the potential crosses successfully produced offspring (Table 6). In some cases, certain between‐population pairings resulted in predation by one individual on their potential mate; this was never observed with any of the within‐population pairings. Despite amplexus being observed in all treatment groups, none of the heterospecific pairings involving *H. texana* or SMS *Hyalella* sp. produced any offspring. This observation is consistent with those two populations being completely isolated reproductively from all other tested populations.

**Table 6:** Cumulative percentage of crosses that successfully produced offspring after 8 weeks, with number of replications in parentheses (which was sometimes limited by the availability of individuals size‐matched for compatible pairings)

	*Hyalella texana*	Devils	Comal	SMR	SMS
*H. texana*	100% (4)				
Devils	0% (6)	100% (3)			
Comal	0% (5)	75% (4)	100% (4)		
SMR	0% (3)	100% (3)	75% (4)	100% (3)	
SMS	0% (3)	0% (4)	0% (4)	0% (3)	100% (4)

Diagonal represents same‐population controls.

**Figure 5:**
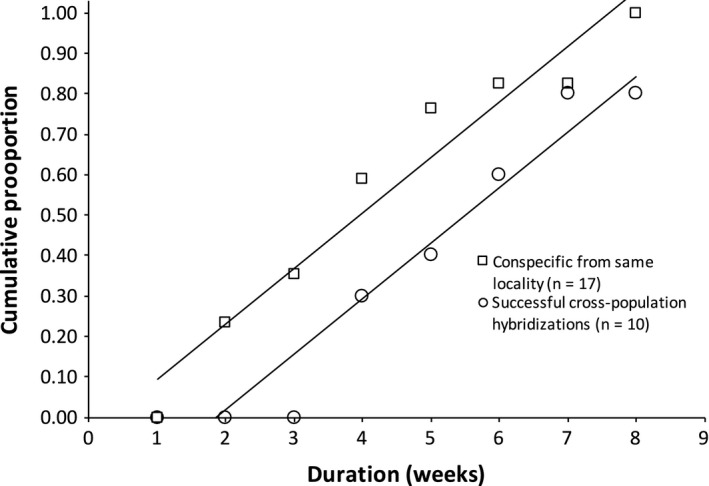
Cumulative proportion of successfully reproducing pairs across time. By the second week, conspecific pairs had produced offspring; however, none of the heterospecific crosses produced offspring until at least 4 weeks had elapsed. Only the heterospecific crosses that successfully produced offspring are depicted. None of the heterospecific pairings including *H. texana* or SMS *Hyalella* sp. successfully produced offspring

Among the replicates that successfully produced offspring, there was noticeable resistance by the heterospecific pairs to mate. Conspecific control pairs produced offspring as early as 2 weeks into mating trials while none of the successful heterospecific pairs produced offspring until after at least 4 weeks (Figure 5). This result is consistent with interfertile heterospecific populations having some degree of prezygotic reproductive isolation. After rearing hybrid offspring to adulthood, all hybrid offspring successfully produced offspring suggesting that hybrids are fertile.

### 3.4 Evaluation of factors contributing to evolution of reproductive isolation

**Figure 6:**
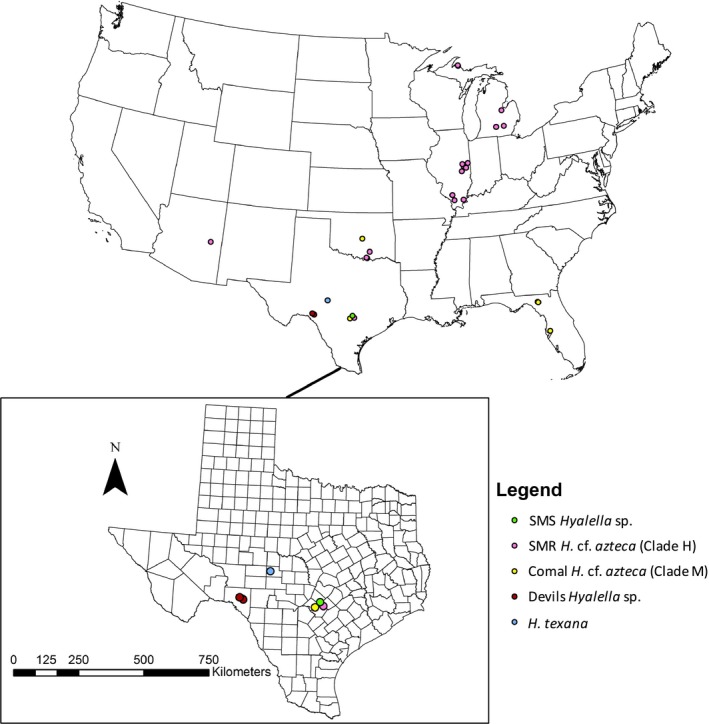
Geographic distribution of clades inferred through genetic analysis for which reproductive isolation was assessed. Note that the populations found to be reproductively isolated occur at only a single locality each while the interfertile populations belong to widely distributed clades

Reproductive isolation was not found to be significantly explainable by genetic distance (*r *=* *0.54, *p *=* *.16). However, geography was found to be an important factor (Figure 6) as the number of populations of each clade and the length of reach occupied by each population was both found to significantly explain the occurrence of reproductive isolation (Figure 6, Table 7).

**Table 7:** Results of ANOVAs on the relationship between geography and reproductive isolation

Model	*df*	*F* ratio	*p*
River kM	4	5.91E+32	≪.001
*n* of localities	4	5.21E+32	≪.001
Interaction (*n:*kM)	4	4.46E+32	≪.001

Geography in both size of distribution and number of known localities for each haplotype was found to significantly explain the occurrence of reproductive isolation.

## 4 DISCUSSION

Appreciable morphological and molecular differentiation were observed in the five populations of *Hyalella* in this study (Figure 1, Figure 3). The molecular analysis suggests that (i) the five populations in this study along with numerous other nominal *Hyalella* populations represent a polytomy with deep divergence, and (ii) *H. *cf. *azteca* is a paraphyletic complex of populations with appreciable molecular divergence between several distinct lineages despite fitting the usual expectations of a morphological species (Figure 1, Witt & Hebert, 2000; Witt et al., 2006; Wellborn & Broughton, 2008; Dionne et al., 2011). Based on the depth of molecular divergence between populations, and the paraphyletic distribution of populations conforming to the *H. *cf. *azteca* morphotype, it is likely that this morphology is due to selection more so than common ancestry; although it is unclear if selection is stabilizing, causing the retention of ancestral morphology, or directional causing convergence. The observation of morphological diversity not conforming to an inferred history of shared common ancestry is not unique to *Hyalella* (Faria et al., 2014; McGee, Neches, & Seehausen, 2016).

The common‐garden populations experienced some degree of morphological differentiation from wild‐caught ancestors after just five generations in captivity (Table 5). It is likely that differentiation of lab stock occurred via drift or plasticity due to inevitable bottlenecks when establishing populations in captivity, as morphological divergence and local adaptation have been shown to occur rapidly in captivity (Fragata et al., 2014). However, differentiation was convergent toward the *H. *cf. *azteca* morphotype which could explain the pervasiveness of this form.

Only some of the populations were found to be interfertile and this did not strongly correlate with history of common ancestry or morphological similarity (Figure 4). The three interfertile populations were interfertile with each other in all possible combinations but never produced offspring with either of the reproductively isolated populations. The two reproductively isolated populations were shown to be completely reproductively isolated from all three of the interfertile populations as well as from each other. At this time, the mechanism of reproductive isolation is unknown although amplexus was observed in all combinations, suggesting that the mode of reproductive isolation is gametic or postzygotic for the completely reproductively isolated populations, or at least not entirely behavioral. However, all of the heterospecific mating trials showed evidence of reproductive isolation, including interfertile combinations (Figure 5). This finding demonstrates viable hybridization between morphologically distinct populations and presents evidence of behavioral prezygotic reproductive isolation between populations of what were formerly considered populations of the *H. *cf. *azteca* cryptic species complex.

Spring‐endemic populations are thought to have evolved spring‐specific adaptations in geographic isolation during the droughts of the Holocene (Al‐Rabab'ah & Williams, 2004; Davis & Shaw, 2001; Ellwood & Gose, 2006; Hall & Penner, 2013; Nordt, Boutton, Hallmark, & Waters, 1994; Russ, Loyd, & Boutton, 2000). Therefore, divergence between populations likely occurred in the absence of gene flow; thus, sympatrically occurring populations likely represent secondary contact. It is unclear if divergence occurred directionally due to selection or drift during periods of geographic isolation. However, molecular distance did not predict morphology or reproductive isolation, but geographic range size was found to be negatively correlated with interfertility (Figure 6, Table 7). This result is consistent with the hypothesis that geographic isolation drives accelerated divergence (Woolfit & Bromham, 2005) as spring‐endemic populations were both found to be completely reproductively isolated and morphologically distinct. Shared morphology among spring populations despite relatively large genetic distance suggests that similar gradients of selection operate in different spring habitats driving convergence of spring‐adapted *Hyalella*. The finding of morphologically divergent *Hyalella* endemic to springs is not unique to this study (Cole & Watkins, 1977; Stevenson & Peden, 1973), and repeated parallel adaptations have been shown to occur when selection is strong and similar across different populations (Butlin et al., 2014; Eroukhmanoff et al., 2009; Westram et al., 2016). It is possible that the ecological gradient that is responsible for morphological divergence in spring *Hyalella* is also associated with the evolution of reproductive isolation (Rundle & Nosil, 2005). If so, *Hyalella*, particularly spring‐endemic *Hyalella*, may represent a good model for the study of ecological speciation.

It is remarkable that *Hyalella* was recovered as a monophyletic taxon as the depth of divergence between different *Hyalella* lineages is comparable to the depth of divergence observed among the other amphipod families included in our analysis (depth of divergence between outgroups in Figure 1 is comparable to the divergence found within *Hyalella*). However, this could be due to a generation time effect leading to different rates of molecular divergence (Chao & Carr, 1993; Ohta, 1993; Thomas, Welch, Lanfear, & Bromham, 2010) as *Hyalella* has roughly four generations per year—a much faster rate of reproduction than observed in the other amphipod families discussed herein (Crawford & Tarter, 1979; Welton, 1979).

We also only used a single mitochondrial locus because of the abundance of archived COI sequences for amphipods; however, the rate of divergence may be too rapid at the COI locus to properly estimate relationships with such deep divergence (Figure 2). It is possible that divergence between lineages is approaching saturation which appears to have occurred around 0.15 subst./site (Figure 2). However, separate analyses looking at each codon position revealed that first and second codon positions account for observed saturation of the COI locus while the third position conforms to the expectations of neutral evolution (Figure S2). A comparison of the amount of observed pairwise substitutions indicates that the third codon position is evolving approximately 2.65 to 3 times faster than the second and first codon positions, respectively (Figure S3).

It is important to point out that the present study recovered fewer haplotypes than previous authors despite sequencing the same locus and using the same sequences published by other authors on GenBank. This is likely due to the trimming of sequences to much fewer base pairs in order to have a complete alignment as different authors amplified different regions of the COI locus. Therefore, it is likely that variable sites were eliminated that other authors used to identify haplotypes.

Divergence in isolation may lead to scenarios that allow for trait deterioration due to genetic drift (Bromham, 2009; Woolfit & Bromham, 2005), including attributes that affect reproductive isolation. Presumably, there is strong stabilizing selection within a population to maintain interfertility with other members of the same population. In smaller populations, individuals that are divergent in reproductive compatibility have a greater proportional effect on the gene pool of the population (Gillespie, 2001; Woolfit & Bromham, 2005). Therefore, it is more likely that genomic changes that lead to barriers to interfertility will be retained in smaller populations. It is also less likely for larger populations to diverge from the reproductive type of ancestral populations if they experience stabilizing selection for interfertility due to lower susceptibility to drift in larger populations. Therefore, abundant and widespread taxa experiencing stabilizing selection may maintain interfertility with many different lineages, especially other widespread taxa, while local endemics experience drift or divergent selection. The experimental observations presented herein are consistent with this hypothesis, but it requires further investigation. Identifying divergent loci associated with reproductive isolation could shed light on the factors that contribute to the evolution of reproductive isolation.

## AUTHORS CONTRIBUTIONS

M. L. D. Worsham involved in research design, field collections, laboratory experiments, molecular phylogeny, statistical analysis, manuscript preparation, and maps. E. P. Julius involved in field collections and laboratory experiments. C. C. Nice involved in molecular phylogeny, statistical analysis, and manuscript preparation. P. H. Diaz involved in multivariate statistics and maps. D. G. Huffman involved in field collections, material support and manuscript preparation.

## DATA ACCESSIBILITY

Genbank accessions: see Table S5.
