# Supplementary material for: Geographic isolation facilitates the evolution of reproductive isolation and morphological divergence
Source: Ecol Evol. 2017 Oct 27;7(23):10278–88. doi: 10.1002/ece3.3474 (PMC5723600; doi:10.1002/ece3.3474)
Supplement: Supplementary file 1 [file ECE3-7-10278-s001.docx]

# Supporting Information

Figure S1.) Demonstration of the various morphometric measurements. Total length was taken at the midline of individuals because this was found to not vary in length regardless of the curvature of the amphipods body.

To study the effect that different rates of evolution at different codon positions could be having on saturation curves, the overall sequence alignment was parsed into three separate alignments, one for each codon position. MrBayes was used to estimate a phylogeny for each codon position using the TVM + I + G model for first and second codon positions and TVM + G for third codon positions. Bayesian model corrected pairwise distances were compared to observed substitutions to generate a saturation plot for each codon position (Figure S2). Observed pairwise distances for each codon positon were also plotted against each other to compare relative rates of evolution at each position (Figure S3).

Figure S2.) A.) Saturation plot at first codon positions, B.) saturation plot at second codon positions, C.) saturation plot at third codon positions.

Figure S3.) A.) Comparison of observed pairwise substitutions at first and second codon positons, B.) comparison of observed pairwise substitutions at second and third codon positons, C.) comparison of observed pairwise substitutions at first and third codon positons. Black regression line represents theoretically equal rates of evolution; red regression line represents observed relationships.

To examine placement of hypothesized groups with morphological data, linear discriminant function analysis (LDA) was used in R using the lda (Venables and Ripley 2002) function in the “MASS” package. Morphological data was transformed with log(X+1) prior to analysis. A leave-one-out cross validation test was performed to examine validity of the original model. Wilks lambda was calculated to determine significance in the means of the different groups.

The first axis of the LDA explained 91% of the variance with the second axis explaining 7%. Classification results from LDA showed that predicted groups were classified at 83% for the original model and for the jackknifed model (Table S1). Discriminant function coefficients for spine count had the largest loadings on axis I while longest spine had the highest loadings for axis II (Table S2). There was a significant difference among the predicted groups with the measured morphological characteristics (P < 0.0001; λ = 0.0018).

Table S1.) Classification results from linear discriminant analysis using morphometric data from five populations each having 20 individuals from wild-caught amphipods. Note that all four of the *H. texana* that were misclassified as SMS *Hyalella* sp. were juveniles.

|  | **SMS** | **Devils** | **Comal** | ***H. texana*** | **SMR** |
| --- | --- | --- | --- | --- | --- |
| **SMS** | 20 | 0 | 0 | 0 | 0 |
| **Devils** | 0 | 15 | 5 | 0 | 0 |
| **Comal** | 0 | 1 | 15 | 0 | 4 |
| ***H. texana*** | 4 | 0 | 0 | 16 | 0 |
| **SMR** | 0 | 0 | 3 | 0 | 17 |
| **Totals** | **24** | **16** | **23** | **16** | **21** |

Table S2.) Discriminant function coefficients from linear discriminant analysis performed on morphological data from wild-caught amphipods for five experimental populations.

| **Variable** | **DA I** | **DA II** |
| --- | --- | --- |
| Longest Spine | -6.671 | 4.794 |
| Spine Count | -13.876 | -43.128 |
| Head / Total Length | -28.921 | 10.718 |

Table S3.) Results of neonate growth rate study.

| **Population** | **Brood Size** | **Mean neonate length (mm)** | **Slope (growth rate)** | **R^2^** | **Age of Sexual Dimorphism (weeks)** | **Mean Length at Sexual Dimorphism** |
| --- | --- | --- | --- | --- | --- | --- |
| Comal | 5 | 1.292 | 0.59 | 0.5 | 3 | 2.96 |
| Comal | 4 | 1.021 | 0.89 | 0.93 | 2 | 2.92 |
| Devils | 24 | 1.201 | 0.45 | 0.63 | 4 | 3.12 |
| Devils | 22 | 1.282 | 0.76 | 0.77 | 3 | 3.00 |
| Devils | 12 | 1.416 | 0.65 | 0.85 | 3 | 3.34 |
| *H. texana* | 11 | 1.071 | 1.52 | 0.8 | 3 | 4.89 |
| *H. texana* | 7 | 1.097 | 1.1 | 0.91 | 3 | 4.66 |
| *H. texana* | 8 | 1.118 | 0.73 | 0.94 | 3 | 3.31 |
| SMR | 6 | 0.966 | 0.26 | 0.55 | 3 | 3.15 |
| SMR | 11 | 1.252 | 0.3 | 0.57 | 3 | 2.45 |
| SMR | 5 | 1.024 | 0.29 | 0.69 | 3 | 1.8 |
| SMS | 6 | 1.185 | 0.74 | 0.92 | 3 | 3.39 |
| SMS | 6 | 1.246 | 0.62 | 0.83 | 4 | 3.79 |
| SMS | 7 | 1.265 | 0.66 | 0.94 | 4 | 3.44 |

Table S4.) Results of neonate growth rate analysis. Age and length at which neonates became sexually dimorphic did varied across the populations significantly though growth rate did not.

| **SOV** | **Response Variable** | ***df*** | ***F* ratio** | ***p*** |
| --- | --- | --- | --- | --- |
| Population | Growth rate | 4 | 3.236 | 0.084 |
| Population | Age of dimorphism | 4 | 14.39 | 0.0017 |
| Population | Length of dimorphism | 4 | 28.39 | 0.0002 |

Table S5.) List of sequences and associated GenBank accession numbers. Clade, haplotype, and geographic information included where applicable.

| **GenBank Accession** | **Clade** | **Haplotype** | **Geographic Location** | **Latitude** | **Longitude** | **Author(s)** |
| --- | --- | --- | --- | --- | --- | --- |
| DQ464668 | Clade A | 48 | White River, Nevada | 37.4605556 | -115.1933333 | Witt et al. 2006 |
| DQ464666 | Clade A | 49 | Steptoe Valley Spring, Nevada | 40.2211111 | -114.7475 | Witt et al. 2006 |
| DQ464667 | Clade A | 49 | Steptoe Valley Spring, Nevada | 40.2211111 | -114.7475 | Witt et al. 2006 |
| DQ464671 | Clade B | 46 | Grass Spring, Nevada | 39.5186111 | -114.9138889 | Witt et al. 2006 |
| DQ464672 | Clade B | 46 | Grass Spring, Nevada | 39.5186111 | -114.9138889 | Witt et al. 2006 |
| DQ464673 | Clade B | 46 | Grass Spring, Nevada | 39.5186111 | -114.9138889 | Witt et al. 2006 |
| DQ464674 | Clade B | 46 | Grass Spring, Nevada | 39.5186111 | -114.9138889 | Witt et al. 2006 |
| DQ464669 | Clade B | 47 | Billy Pope Spring, Nevada | 39.6277778 | -114.905 | Witt et al. 2006 |
| DQ464670 | Clade B | 47 | Billy Pope Spring, Nevada | 39.6277778 | -114.905 | Witt et al. 2006 |
| AY152792 | Clade C | 86 | Bubbling Springs, Arizona | 34.7733333 | -111.9016667 | Witt et al. 2003 |
| AY152793 | Clade C | 87 | Bubbling Springs, Arizona | 34.7733333 | -111.9016667 | Witt et al. 2003 |
| DQ464691 | Clade D | 39 | Lower Vine Ranch Spring, Ash Meadows, Nevada | 37.0183333 | -117.3866667 | Witt et al. 2006 |
| DQ464692 | Clade D | 39 | Lower Vine Ranch Spring, Ash Meadows, Nevada | 37.0183333 | -117.3866667 | Witt et al. 2006 |
| DQ464693 | Clade D | 39 | Lower Vine Ranch Spring, Ash Meadows, Nevada | 37.0183333 | -117.3866667 | Witt et al. 2006 |
| DQ464690 | Clade D | 40 | Surprise Spring, Ash Meadows, Nevada | 37.0005556 | -117.3427778 | Witt et al. 2006 |
| DQ464689 | Clade D | 41 | Antelope Spring, California | 37.3311111 | -118.0894444 | Witt et al. 2006 |
| DQ464687 | Clade D | 41 | Spring 94, California | 37.1938889 | -118.1991667 | Witt et al. 2006 |
| DQ464688 | Clade D | 41 | Spring 94, California | 37.1938889 | -118.1991667 | Witt et al. 2006 |
| DQ464683 | Clade D | 41 | Spring 97, California | 37.2977778 | -118.1947222 | Witt et al. 2006 |
| DQ464684 | Clade D | 41 | Spring 97, California | 37.2977778 | -118.1947222 | Witt et al. 2006 |
| DQ464685 | Clade D | 41 | Spring 97, California | 37.2977778 | -118.1947222 | Witt et al. 2006 |
| DQ464686 | Clade D | 41 | Spring 97, California | 37.2977778 | -118.1947222 | Witt et al. 2006 |
| DQ464705 | Clade E | 31 | Mule Spring, California | 37.1061111 | -118.2008333 | Witt et al. 2006 |
| DQ464706 | Clade E | 31 | Mule Spring, California | 37.1061111 | -118.2008333 | Witt et al. 2006 |
| DQ464709 | Clade E | 31 | Owens River; Lubkin Creek, California | 36.5744444 | -118.0097222 | Witt et al. 2006 |
| DQ464710 | Clade E | 31 | Owens River; Lubkin Creek, California | 36.5744444 | -118.0097222 | Witt et al. 2006 |
| DQ464707 | Clade E | 31 | Spring 103, California | 37.4927778 | -118.3325 | Witt et al. 2006 |
| DQ464712 | Clade E | 31 | Tuttle Creek, California | 36.5719444 | -118.1080556 | Witt et al. 2006 |
| DQ464713 | Clade E | 31 | Tuttle Creek, California | 36.5719444 | -118.1080556 | Witt et al. 2006 |
| DQ464714 | Clade E | 31 | Tuttle Creek, California | 36.5719444 | -118.1080556 | Witt et al. 2006 |
| DQ464715 | Clade E | 31 | Tuttle Creek, California | 36.5719444 | -118.1080556 | Witt et al. 2006 |
| DQ464711 | Clade E | 32 | Lubkin Creek, California | 36.5419444 | -118.0602778 | Witt et al. 2006 |
| DQ464708 | Clade E | 33 | Warm Spring, California | 37.2666667 | -118.2708333 | Witt et al. 2006 |
| DQ464704 | Clade E | 34 | Big Spring, California | 37.75 | -118.9383333 | Witt et al. 2006 |
| GU066812 | Clade F | 19 | Yellowstone Lake, Wyoming | 44.4182444 | -110.54135 | Lovalvo et al. 2010 |
| DQ464698 | Clade F | 37 | Side Hill Spring, Nevada | 38.2558333 | -116.6886111 | Witt et al. 2006 |
| DQ464699 | Clade F | 37 | Side Hill Spring, Nevada | 38.2558333 | -116.6886111 | Witt et al. 2006 |
| DQ464694 | Clade F | 38 | Fish Slough, California | 37.5169444 | -118.3997222 | Witt et al. 2006 |
| DQ464695 | Clade F | 38 | Fish Slough, California | 37.5169444 | -118.3997222 | Witt et al. 2006 |
| DQ464696 | Clade F | 38 | Fish Slough, California | 37.5169444 | -118.3997222 | Witt et al. 2006 |
| DQ464697 | Clade F | 38 | Fish Slough, California | 37.5169444 | -118.3997222 | Witt et al. 2006 |
| AY152779 | Clade F | 74 | Black River, Arizona | 33.8516667 | -109.315 | Witt et al. 2003 |
| AY152780 | Clade F | 75 | Black River, Arizona | 33.8516667 | -109.315 | Witt et al. 2003 |
| AY152781 | Clade F | 76 | Black River, Arizona | 33.8516667 | -109.315 | Witt et al. 2003 |
| AY152782 | Clade F | 77 | Black River, Arizona | 33.8516667 | -109.315 | Witt et al. 2003 |
| AY152783 | Clade F | 78 | Black River, Arizona | 33.8516667 | -109.315 | Witt et al. 2003 |
| AY152784 | Clade F | 79 | Black River, Arizona | 33.8516667 | -109.315 | Witt et al. 2003 |
| AY152785 | Clade F | 80 | Montezuma Well, Arizona | 34.65 | -111.75 | Witt et al. 2003 |
| AY152786 | Clade F | 81 | Montezuma Well, Arizona | 34.65 | -111.75 | Witt et al. 2003 |
| AY152787 | Clade F | 82 | Montezuma Well, Arizona | 34.65 | -111.75 | Witt et al. 2003 |
| AY152788 | Clade F | 83 | Montezuma Well, Arizona | 34.65 | -111.75 | Witt et al. 2003 |
| AY152789 | Clade F | 83 | Montezuma Well, Arizona | 34.65 | -111.75 | Witt et al. 2003 |
| AY152790 | Clade F | 84 | Montezuma Well, Arizona | 34.65 | -111.75 | Witt et al. 2003 |
| AY152795 | Clade F | 89 | Crescent Lake, Arizona | 33.9166667 | -109.4233333 | Witt et al. 2003 |
| AY152796 | Clade F | 90 | Crescent Lake, Arizona | 33.9166667 | -109.4233333 | Witt et al. 2003 |
| KF596727 | Clade G | 2 | Blodgett Reservoir, California | 38.5176 | -121.2109 | Weston et al. 2013 |
| KF596728 | Clade G | 2 | Blodgett Reservoir, California | 38.5176 | -121.2109 | Weston et al. 2013 |
| KF596729 | Clade G | 2 | Blodgett Reservoir, California | 38.5176 | -121.2109 | Weston et al. 2013 |
| KF596747 | Clade G | 2 | Pleasant Grove Creek, California | 38.8052 | -121.3069 | Weston et al. 2013 |
| EU621744 | Clade G | 22 | Eel Lake, Oregon | 43.6066111 | -124.1733361 | Wellborn and Broughton 2008 |
| EU621742 | Clade G | 22 | Siltcoos Lake, Oregon | 43.8767056 | -124.0874167 | Wellborn and Broughton 2008 |
| JX446329 | Clade H | 16 | Clear Pond; Japan House Pond, Illinois | 40.1381 | -87.7417 | Major et al. 2013 |
| JX446323 | Clade H | 16 | Eagle Creek, Illinois | 37.6558 | -88.3743 | Major et al. 2013 |
| JX446338 | Clade H | 16 | Eagle Creek, Illinois | 37.6558 | -88.3743 | Major et al. 2013 |
| JX446327 | Clade H | 16 | East Fork Embarras River, Illinois | 39.9451 | -88.1231 | Major et al. 2013 |
| JX446324 | Clade H | 16 | Flat Branch, Illinois | 39.5997 | -88.3214 | Major et al. 2013 |
| JX446325 | Clade H | 16 | Flat Branch, Illinois | 39.5997 | -88.3214 | Major et al. 2013 |
| JX446326 | Clade H | 16 | Flat Branch, Illinois | 39.5997 | -88.3214 | Major et al. 2013 |
| JX446319 | Clade H | 16 | Indian Creek, Illinois | 37.6557 | -89.1798 | Major et al. 2013 |
| JX446320 | Clade H | 16 | Indian Creek, Illinois | 37.6557 | -89.1798 | Major et al. 2013 |
| JX446337 | Clade H | 16 | Indian Creek, Illinois | 37.6557 | -89.1798 | Major et al. 2013 |
| JX446333 | Clade H | 16 | Ives Lake, Michigan | 46.8437 | -87.8548 | Major et al. 2013 |
| JX446334 | Clade H | 16 | Ives Lake, Michigan | 46.8437 | -87.8548 | Major et al. 2013 |
| JX446330 | Clade H | 16 | Japan House Pond, Illinois | 40.0932 | -88.2168 | Major et al. 2013 |
| JX446321 | Clade H | 16 | Panther Creek, Illinois | 38.0235 | -89.3337 | Major et al. 2013 |
| JX446322 | Clade H | 16 | Panther Creek, Illinois | 38.0235 | -89.3337 | Major et al. 2013 |
| JX446335 | Clade H | 16 | Pine River, Michigan | 46.8829 | -87.869 | Major et al. 2013 |
| JX446336 | Clade H | 16 | Pine River, Michigan | 46.8829 | -87.869 | Major et al. 2013 |
| JX446328 | Clade H | 16 | Pope Branch; East Fork Embarras River, Illinois | 39.8141 | -87.9273 | Major et al. 2013 |
| JX446331 | Clade H | 16 | Tittabawasee River, Michigan | 43.4812 | -84.0924 | Major et al. 2013 |
| JX446332 | Clade H | 16 | Tittabawasee River, Michigan | 43.4812 | -84.0924 | Major et al. 2013 |
| EU621751 | Clade H | 20 | Sullivan Lake, Michigan | 42.3991694 | -84.0567194 | Wellborn and Broughton 2008 |
| EU621754 | Clade H | 20 | Sullivan Lake, Michigan | 42.3991694 | -84.0567194 | Wellborn and Broughton 2008 |
| EU621748 | Clade H | 21 | Blue River, Oklahoma | 34.3196611 | -96.5978306 | Wellborn and Broughton 2008 |
| EU621745 | Clade H | 21 | Briar Creek, Oklahoma | 33.914547 | -96.869446 | Wellborn and Broughton 2008 |
| EU621750 | Clade H | 21 | Duck Lake, Michigan | 42.389075 | -84.7850583 | Wellborn and Broughton 2008 |
| MF990283 | Clade H | 21 | San Marcos River, Texas | 29.89095 | -97.932425 | Present study |
| AY152797 | Clade H | 91 | Rainbow Lake, Arizona | 34.1583333 | -109.9816667 | Witt et al. 2003 |
| KF596737 | Clade I | 5 | Laguna Lake, California | 38.2064 | -122.7636 | Weston et al. 2013 |
| KF596738 | Clade I | 5 | Laguna Lake, California | 38.2064 | -122.7636 | Weston et al. 2013 |
| KF596736 | Clade I | 6 | Grayson Creek, California | 37.9665 | -122.0666 | Weston et al. 2013 |
| JX446339 | Clade J | 13 | Clear Pond, Illinois | 40.1381 | -87.7417 | Major et al. 2013 |
| JX446340 | Clade J | 13 | Clear Pond, Illinois | 40.1381 | -87.7417 | Major et al. 2013 |
| EU621755 | Clade J | 13 | Duck Lake, Michigan | 42.389075 | -84.7850583 | Wellborn and Broughton 2008 |
| JX446342 | Clade J | 13 | Ives Lake, Michigan | 46.8437 | -87.8548 | Major et al. 2013 |
| JX446351 | Clade J | 13 | La Crosse River, Wisconsin | 43.9008 | -90.9901 | Major et al. 2013 |
| JX446352 | Clade J | 13 | La Crosse River, Wisconsin | 43.9008 | -90.9901 | Major et al. 2013 |
| EU621758 | Clade J | 13 | Long Lake, Michigan | 45.2020528 | -83.4677083 | Wellborn and Broughton 2008 |
| JX446343 | Clade J | 13 | Middle (2nd) Pine Lake, Michigan | 46.8698 | -87.8562 | Major et al. 2013 |
| JX446344 | Clade J | 13 | Pine River, Michigan | 46.8829 | -87.869 | Major et al. 2013 |
| JX446345 | Clade J | 13 | Pine River; Middle (2nd) Pine Lake, Michigan | 46.8698 | -87.8562 | Major et al. 2013 |
| EU621762 | Clade J | 13 | Sullivan Lake, Michigan | 42.3991694 | -84.0567194 | Wellborn and Broughton 2008 |
| JX446348 | Clade J | 13 | Tittabawasee River, Michigan | 43.4812 | -84.0924 | Major et al. 2013 |
| EU621760 | Clade J | 13 | Turner Lake, Michigan | 41.8230778 | -84.8092111 | Wellborn and Broughton 2008 |
| JX446349 | Clade J | 13 | Valens Reservoir, Ontario | 43.3867 | -80.1333 | Major et al. 2013 |
| JX446353 | Clade J | 13 | Winnebago County Lake, Wisconsin | 44.1126 | -88.3253 | Major et al. 2013 |
| JX446354 | Clade J | 13 | Winnebago County Lake, Wisconsin | 44.1126 | -88.3253 | Major et al. 2013 |
| JX446350 | Clade J | 14 | Tittabawasee River, Michigan | 43.4812 | -84.0924 | Major et al. 2013 |
| JX446341 | Clade J | 15 | Ives Lake; Middle (2nd) Pine Lake, Michigan | 46.8437 | -87.8548 | Major et al. 2013 |
| JX446366 | Clade K | 9 | Pine Lake, Michigan | 46.8803 | -87.8679 | Major et al. 2013 |
| JX446365 | Clade K | 9 | Pine Lake; Middle (2nd) Pine Lake, Michigan | 46.8698 | -87.8562 | Major et al. 2013 |
| EU621740 | Clade K | 10 | George Pond, Michigan |  |  | Wellborn and Broughton 2008 |
| AJ968918 | Clade K | 10 | Green Lake, Wisconsin | 43.8377917 | -88.9666 | Galbreath et al. 2009 |
| JX446356 | Clade K | 10 | Ives Lake, Michigan | 46.8437 | -87.8548 | Major et al. 2013 |
| JX446359 | Clade K | 10 | Ives Lake, Michigan | 46.8437 | -87.8548 | Major et al. 2013 |
| JX446360 | Clade K | 10 | Ives Lake, Michigan | 46.8437 | -87.8548 | Major et al. 2013 |
| AJ968916 | Clade K | 10 | Lake Beulah, Wisconsin | 42.8284639 | -88.3790694 | Galbreath et al. 2009 |
| JX446357 | Clade K | 10 | Middle (2nd) Pine Lake, Michigan | 46.8698 | -87.8562 | Major et al. 2013 |
| EU621738 | Clade K | 10 | Otis Marsh, Michigan | 41.5966167 | -86.9246 | Wellborn and Broughton 2008 |
| JX446361 | Clade K | 10 | Trout Lake Drainage, Michigan | 46.8534 | -87.8884 | Major et al. 2013 |
| JX446362 | Clade K | 10 | Winnebago County Lake, Wisconsin | 44.1126 | -88.3253 | Major et al. 2013 |
| JX446363 | Clade K | 10 | Winnebago County Lake, Wisconsin | 44.1126 | -88.3253 | Major et al. 2013 |
| JX446358 | Clade K | 11 | Pine Lake, Michigan | 46.8803 | -87.8679 | Major et al. 2013 |
| JX446355 | Clade K | 12 | Rio Cebolla Stream, New Mexico | 35.8531 | -106.7633 | Major et al. 2013 |
| DQ464664 | Clade K | 23 | Hot Spring, Nevada | 38.3772222 | -115.1475 | Witt et al. 2006 |
| DQ464665 | Clade K | 23 | Hot Spring, Nevada | 38.3772222 | -115.1475 | Witt et al. 2006 |
| EU621736 | Clade K | 23 | Lost Lake, Oregon | 45.4915889 | -121.8208444 | Wellborn and Broughton 2008 |
| DQ464662 | Clade K | 23 | Meadow Valley Wash, Nevada | 37.6530556 | -114.5005556 | Witt et al. 2006 |
| DQ464663 | Clade K | 23 | Meadow Valley Wash, Nevada | 37.6530556 | -114.5005556 | Witt et al. 2006 |
| EU621733 | Clade K | 23 | Suttle Lake, Oregon | 44.4250722 | -121.7329222 | Wellborn and Broughton 2008 |
| DQ464661 | Clade K | 50 | Meadow Valley Wash, Nevada | 37.6530556 | -114.5005556 | Witt et al. 2006 |
| DQ464660 | Clade K | 51 | Comins Lake, Nevada | 39.165 | -114.8116667 | Witt et al. 2006 |
| AY152791 | Clade K | 85 | Marshall Lake, Arizona | 35.1186389 | -111.5364167 | Witt et al. 2003 |
| AY152794 | Clade K | 88 | Comins Lake, Nevada | 39.165 | -114.8116667 | Witt et al. 2003 |
| AY152798 | Clade K | 92 | Rainbow Lake, Arizona | 34.1583333 | -109.9816667 | Witt et al. 2003 |
| AY152799 | Clade K | 93 | Unnamed Pond, Arizona | 34.0666667 | -109.5516667 | Witt et al. 2003 |
| AY152800 | Clade K | 94 | Unnamed Pond, Arizona | 34.0666667 | -109.5516667 | Witt et al. 2003 |
| AY152801 | Clade K | 95 | Unnamed Pond, Arizona | 34.0666667 | -109.5516667 | Witt et al. 2003 |
| AY152802 | Clade K | 96 | Unnamed Pond, Arizona | 34.0666667 | -109.5516667 | Witt et al. 2003 |
| AY152803 | Clade K | 96 | Unnamed Pond, Arizona | 34.0666667 | -109.5516667 | Witt et al. 2003 |
| AY152804 | Clade K | 97 | Unnamed Pond, Arizona | 34.0666667 | -109.5516667 | Witt et al. 2003 |
| EU621729 | Clade L | 7 | Chief Noonday Lake, Michigan | 42.6411361 | -85.5077917 | Wellborn and Broughton 2008 |
| EU621732 | Clade L | 7 | Duck Lake, Michigan | 42.389075 | -84.7850583 | Wellborn and Broughton 2008 |
| JX446367 | Clade L | 7 | Joe’s Pond, Vermont | 44.4069 | -72.2203 | Major et al. 2013 |
| AJ968915 | Clade L | 7 | Lake Beulah, Wisconsin | 42.8284639 | -88.3790694 | Galbreath et al. 2009 |
| EU621730 | Clade L | 24 | Deep Lake, Michigan | 42.620325 | -85.4584694 | Wellborn and Broughton 2008 |
| AJ968917 | Clade L | 28 | Lake Grada, Blue Mountain, Mississippi | 34.6287222 | -89.0305167 | Galbreath et al. 2009 |
| JX446311 | Clade M | 1 | Lowry Park, Florida | 28.0131 | -82.4664 | Major et al. 2013 |
| JX446314 | Clade M | 1 | Lowry Park, Florida | 28.0131 | -82.4664 | Major et al. 2013 |
| JX446315 | Clade M | 1 | Lowry Park, Florida | 28.0131 | -82.4664 | Major et al. 2013 |
| JX446310 | Clade M | 1 | Peacock Springs, Florida | 30.122 | -83.1322 | Major et al. 2013 |
| MF990282 | Clade M | 17 | Comal River, Texas | 29.7105556 | -98.1276667 | Present study |
| JX446317 | Clade M | 17 | Convict Spring, Florida | 30.0884 | -83.0959 | Major et al. 2013 |
| EU621728 | Clade M | 17 | Lake Thunderbird, Oklahoma | 35.2269444 | -97.2433917 | Wellborn and Broughton 2008 |
| JX446316 | Clade M | 17 | Lowry Park, Florida | 28.0131 | -82.4664 | Major et al. 2013 |
| JX446312 | Clade M | 18 | Lowry Park, Florida | 28.0131 | -82.4664 | Major et al. 2013 |
| JX446313 | Clade M | 18 | Lowry Park, Florida | 28.0131 | -82.4664 | Major et al. 2013 |
| EU621727 | Clade M | 25 | UOBS, Lake Texoma, Oklahoma | 33.8798917 | -96.7987583 | Wellborn and Broughton 2008 |
| DQ464650 | Clade N | 3 | Afton Canyon Spring; Cedar Springs; Mojave River, California | 35.0358333 | -116.3858333 | Witt et al. 2006 |
| DQ464648 | Clade N | 3 | Big Morongo Spring, California | 34.0480556 | -116.5669444 | Witt et al. 2006 |
| DQ464649 | Clade N | 3 | Cedar Springs, California | 34.3088889 | -117.3158333 | Witt et al. 2006 |
| KF596734 | Clade N | 3 | Chualar Creek, California | 36.5583 | -121.5296 | Weston et al. 2013 |
| KF596735 | Clade N | 3 | Grayson Creek, California | 37.9665 | -122.0666 | Weston et al. 2013 |
| DQ464647 | Clade N | 3 | Mojave River, California | 34.5138889 | -117.2613889 | Witt et al. 2006 |
| KF596739 | Clade N | 3 | Morrison Creek, California | 38.5271 | -121.3274 | Weston et al. 2013 |
| KF596740 | Clade N | 3 | Morrison Creek, California | 38.5271 | -121.3274 | Weston et al. 2013 |
| KF596742 | Clade N | 3 | Mosher Slough, California | 38.0325 | -121.3654 | Weston et al. 2013 |
| KF596743 | Clade N | 3 | Mosher Slough, California | 38.0325 | -121.3654 | Weston et al. 2013 |
| KF596744 | Clade N | 3 | Mosher Slough, California | 38.0325 | -121.3654 | Weston et al. 2013 |
| KF596745 | Clade N | 3 | Pleasant Grove Creek, California | 38.8052 | -121.3069 | Weston et al. 2013 |
| KF596746 | Clade N | 3 | Pleasant Grove Creek, California | 38.8052 | -121.3069 | Weston et al. 2013 |
| DQ464644 | Clade N | 3 | Saline Valley Marsh, California | 36.6936111 | -117.8283333 | Witt et al. 2006 |
| DQ464645 | Clade N | 3 | Saline Valley Marsh, California | 36.6936111 | -117.8283333 | Witt et al. 2006 |
| DQ464646 | Clade N | 3 | Saline Valley Marsh, California | 36.6936111 | -117.8283333 | Witt et al. 2006 |
| KF596733 | Clade N | 4 | Chualar Creek, California | 36.5583 | -121.5296 | Weston et al. 2013 |
| KF596741 | Clade N | 4 | Morrison Creek, California | 38.5271 | -121.3274 | Weston et al. 2013 |
| DQ464636 | Clade N | 4 | Warm Springs Canyon, Ash Meadows, Nevada | 35.9677778 | -116.9305556 | Witt et al. 2006 |
| DQ464637 | Clade N | 4 | Warm Springs Canyon, Ash Meadows, Nevada | 35.9677778 | -116.9305556 | Witt et al. 2006 |
| DQ464638 | Clade N | 4 | Warm Springs Canyon, Ash Meadows, Nevada | 35.9677778 | -116.9305556 | Witt et al. 2006 |
| DQ464643 | Clade N | 54 | BLM Spring, California | 37.4805556 | -118.4025 | Witt et al. 2006 |
| DQ464642 | Clade N | 55 | Blue Point Spring, Nevada | 36.3891667 | -114.4322222 | Witt et al. 2006 |
| DQ464641 | Clade N | 56 | Grapevine Spring, Ash Meadows, Nevada | 37.0202778 | -117.3819444 | Witt et al. 2006 |
| DQ464639 | Clade N | 57 | Grapevine Spring, Ash Meadows, Nevada | 37.0202778 | -117.3819444 | Witt et al. 2006 |
| DQ464640 | Clade N | 57 | Grapevine Spring, Ash Meadows, Nevada | 37.0202778 | -117.3819444 | Witt et al. 2006 |
| DQ464634 | Clade N | 57 | Warm Springs Canyon, Ash Meadows, Nevada | 35.9677778 | -116.9305556 | Witt et al. 2006 |
| DQ464635 | Clade N | 57 | Warm Springs Canyon, Ash Meadows, Nevada | 35.9677778 | -116.9305556 | Witt et al. 2006 |
| DQ464624 | Clade P | 62 | Crystal Spring, Ash Meadows, Nevada | 36.4194444 | -116.3238889 | Witt et al. 2006 |
| DQ464625 | Clade P | 62 | Crystal Spring, Ash Meadows, Nevada | 36.4194444 | -116.3238889 | Witt et al. 2006 |
| DQ464626 | Clade P | 62 | Crystal Spring, Ash Meadows, Nevada | 36.4194444 | -116.3238889 | Witt et al. 2006 |
| DQ464623 | Clade P | 63 | Devils Hole, Ash Meadows, Nevada | 36.4252778 | -116.2902778 | Witt et al. 2006 |
| DQ464618 | Clade Q | 65 | North Scruggs Spring, Ash Meadows, Nevada | 36.4327778 | -116.3086111 | Witt et al. 2006 |
| DQ464619 | Clade Q | 65 | North Scruggs Spring, Ash Meadows, Nevada | 36.4327778 | -116.3086111 | Witt et al. 2006 |
| DQ464620 | Clade Q | 65 | North Scruggs Spring, Ash Meadows, Nevada | 36.4327778 | -116.3086111 | Witt et al. 2006 |
| DQ464615 | Clade Q | 66 | Marsh Spring, Ash Meadows, Nevada | 36.4794444 | -116.3258333 | Witt et al. 2006 |
| DQ464616 | Clade Q | 66 | Marsh Spring, Ash Meadows, Nevada | 36.4794444 | -116.3258333 | Witt et al. 2006 |
| DQ464617 | Clade Q | 66 | Marsh Spring, Ash Meadows, Nevada | 36.4794444 | -116.3258333 | Witt et al. 2006 |
| DQ464613 | Clade Q | 66 | Marsh Spring; North and South Indian Springs, Ash Meadows, Nevada | 36.4258333 | -116.3088889 | Witt et al. 2006 |
| DQ464612 | Clade Q | 66 | Marsh Spring; North Indian Spring, Ash Meadows, Nevada | 36.4258333 | -116.3088889 | Witt et al. 2006 |
| DQ464611 | Clade Q | 66 | North and South Indian Springs, Ash Meadows, Nevada | 36.4255556 | -116.3088889 | Witt et al. 2006 |
| DQ464610 | Clade Q | 66 | North Indian Spring, Ash Meadows, Nevada | 36.4258333 | -116.3088889 | Witt et al. 2006 |
| DQ464614 | Clade Q | 67 | Marsh Spring, Ash Meadows, Nevada | 36.4794444 | -116.3258333 | Witt et al. 2006 |
| DQ464632 | Clade O | 58 | Fairbanks Spring, Ash Meadows, Nevada | 36.4902778 | -116.3408333 | Witt et al. 2006 |
| DQ464633 | Clade O | 58 | Fairbanks Spring, Ash Meadows, Nevada | 36.4902778 | -116.3408333 | Witt et al. 2006 |
| DQ464630 | Clade O | 59 | Rogers Spring, Ash Meadows, Nevada | 36.4794444 | -116.3252778 | Witt et al. 2006 |
| DQ464631 | Clade O | 59 | Rogers Spring, Ash Meadows, Nevada | 36.4794444 | -116.3252778 | Witt et al. 2006 |
| AY152805 | *H. montezuma* (Clade K) | 8 | Montezuma Well, Arizona | 34.65 | -111.75 | Witt et al. 2003 |
| AY152806 | *H. montezuma* (Clade K) | 8 | Montezuma Well, Arizona | 34.65 | -111.75 | Witt et al. 2003 |
| AY152807 | *H. montezuma* (Clade K) | 8 | Montezuma Well, Arizona | 34.65 | -111.75 | Witt et al. 2003 |
| DQ464602 | *H. muerta* | 70 | Texas Spring, Death Valley, California | 36.4575 | -116.8369444 | Witt et al. 2006 |
| DQ464600 | *H. muerta* | 70 | Travertine Spring, Death Valley, California | 36.4425 | -116.7769444 | Witt et al. 2006 |
| DQ464601 | *H. muerta* | 70 | Travertine Spring, Death Valley, California | 36.4425 | -116.7769444 | Witt et al. 2006 |
| DQ464603 | *H. muerta* | 70 | Travertine Spring, Death Valley, California | 36.4425 | -116.7769444 | Witt et al. 2006 |
| DQ464681 | *H. sandra* | 42 | Nevares Spring, Death Valley, California | 36.5122222 | -116.8211111 | Witt et al. 2006 |
| DQ464682 | *H. sandra* | 42 | Nevares Spring, Death Valley, California | 36.5122222 | -116.8211111 | Witt et al. 2006 |
| DQ464680 | *H. sandra* | 43 | Nevares Spring, Death Valley, California | 36.5122222 | -116.8211111 | Witt et al. 2006 |
| DQ464679 | *H. sandra* | 44 | Nevares Spring, Death Valley, California | 36.5122222 | -116.8211111 | Witt et al. 2006 |
| DQ464675 | *H. sandra* | 45 | Travertine Spring, Death Valley, California | 36.4425 | -116.7769444 | Witt et al. 2006 |
| DQ464676 | *H. sandra* | 45 | Travertine Spring, Death Valley, California | 36.4425 | -116.7769444 | Witt et al. 2006 |
| DQ464677 | *H. sandra* | 45 | Travertine Spring, Death Valley, California | 36.4425 | -116.7769444 | Witt et al. 2006 |
| DQ464678 | *H. sandra* | 45 | Travertine Spring, Death Valley, California | 36.4425 | -116.7769444 | Witt et al. 2006 |
| MF990280 | *H. texana* | 26 | Clear Creek Springs, Texas | 30.9061667 | -99.9581111 | Present study |
| MF990281 | *H. texana* | 27 | Clear Creek Springs, Texas | 30.9061667 | -99.9581111 | Present study |
| EU621726 | *H. texana* | 27 | Clear Creek Springs, Texas | 30.9061667 | -99.9581111 | Wellborn and Broughton 2008 |
| DQ464724 | n/a | 29 | Lost Creek, Nevada | 36.1561111 | -115.4952778 | Witt et al. 2006 |
| DQ464725 | n/a | 29 | Lost Creek, Nevada | 36.1561111 | -115.4952778 | Witt et al. 2006 |
| DQ464726 | n/a | 29 | Lost Creek, Nevada | 36.1561111 | -115.4952778 | Witt et al. 2006 |
| DQ464727 | n/a | 29 | Lost Creek, Nevada | 36.1561111 | -115.4952778 | Witt et al. 2006 |
| DQ464720 | n/a | 29 | Willow Spring, Nevada | 36.1611111 | -115.4972222 | Witt et al. 2006 |
| DQ464721 | n/a | 29 | Willow Spring, Nevada | 36.1611111 | -115.4972222 | Witt et al. 2006 |
| DQ464722 | n/a | 29 | Willow Spring, Nevada | 36.1611111 | -115.4972222 | Witt et al. 2006 |
| DQ464723 | n/a | 29 | Willow Spring, Nevada | 36.1611111 | -115.4972222 | Witt et al. 2006 |
| DQ464716 | n/a | 30 | Spring 197, Nevada | 38.6361111 | -115.0694444 | Witt et al. 2006 |
| DQ464717 | n/a | 30 | Spring 197, Nevada | 38.6361111 | -115.0694444 | Witt et al. 2006 |
| DQ464718 | n/a | 30 | Spring 197, Nevada | 38.6361111 | -115.0694444 | Witt et al. 2006 |
| DQ464719 | n/a | 30 | Spring 197, Nevada | 38.6361111 | -115.0694444 | Witt et al. 2006 |
| DQ464701 | n/a | 35 | Spring 197, Nevada | 38.6361111 | -115.0694444 | Witt et al. 2006 |
| DQ464702 | n/a | 35 | Spring 197, Nevada | 38.6361111 | -115.0694444 | Witt et al. 2006 |
| DQ464703 | n/a | 35 | Sunny Side Creek, Nevada | 38.4211111 | -115.0744444 | Witt et al. 2006 |
| DQ464700 | n/a | 36 | Steptoe Valley Spring, Nevada | 40.2211111 | -114.7475 | Witt et al. 2006 |
| DQ464656 | n/a | 52 | Hot Creek, Nevada | 38.3772222 | -115.1475 | Witt et al. 2006 |
| DQ464657 | n/a | 52 | Hot Creek, Nevada | 38.3772222 | -115.1475 | Witt et al. 2006 |
| DQ464658 | n/a | 52 | Hot Creek, Nevada | 38.3772222 | -115.1475 | Witt et al. 2006 |
| DQ464659 | n/a | 52 | Hot Creek, Nevada | 38.3772222 | -115.1475 | Witt et al. 2006 |
| DQ464651 | n/a | 53 | Moapa Warm Springs, Nevada | 36.7013889 | -114.7161111 | Witt et al. 2006 |
| DQ464652 | n/a | 53 | Moapa Warm Springs, Nevada | 36.7013889 | -114.7161111 | Witt et al. 2006 |
| DQ464653 | n/a | 53 | Moapa Warm Springs, Nevada | 36.7013889 | -114.7161111 | Witt et al. 2006 |
| DQ464654 | n/a | 53 | Moapa Warm Springs, Nevada | 36.7013889 | -114.7161111 | Witt et al. 2006 |
| DQ464655 | n/a | 53 | Moapa Warm Springs, Nevada | 36.7013889 | -114.7161111 | Witt et al. 2006 |
| DQ464629 | n/a | 60 | Five Springs, Ash Meadows, Nevada | 36.4633333 | -116.3180556 | Witt et al. 2006 |
| DQ464627 | n/a | 61 | Mary Scott Spring, Ash Meadows, Nevada | 36.4366667 | -116.3147222 | Witt et al. 2006 |
| DQ464628 | n/a | 61 | Mary Scott Spring, Ash Meadows, Nevada | 36.4366667 | -116.3147222 | Witt et al. 2006 |
| DQ464621 | n/a | 64 | Point of Rocks Spring, Ash Meadows, Nevada | 36.4022222 | -116.2719444 | Witt et al. 2006 |
| DQ464622 | n/a | 64 | Point of Rocks Spring, Ash Meadows, Nevada | 36.4022222 | -116.2719444 | Witt et al. 2006 |
| DQ464607 | n/a | 68 | North Flagg Spring, Nevada | 38.4238889 | -115.0208333 | Witt et al. 2006 |
| DQ464608 | n/a | 68 | North Flagg Spring, Nevada | 38.4238889 | -115.0208333 | Witt et al. 2006 |
| DQ464609 | n/a | 68 | North Flagg Spring, Nevada | 38.4238889 | -115.0208333 | Witt et al. 2006 |
| DQ464604 | n/a | 69 | Upper White River, Nevada | 38.91 | -115.0597222 | Witt et al. 2006 |
| DQ464605 | n/a | 69 | Upper White River, Nevada | 38.91 | -115.0597222 | Witt et al. 2006 |
| DQ464606 | n/a | 69 | Upper White River, Nevada | 38.91 | -115.0597222 | Witt et al. 2006 |
| MF990278 | n/a | 71 | Devils River, Texas | 29.8995694 | -100.9975472 | Present study |
| MF990279 | n/a | 72 | Devils River, Texas | 29.8995694 | -100.9975472 | Present study |
| MF990277 | n/a | 73 | San Marcos River, Texas | 29.8933611 | -97.9313333 | Present study |
| EF570326 | *Gammarus minus* | n/a | n/a |  |  | Hou et al. 2007 |
| HE860512 | *Metacrangonyx remyi* | n/a | n/a |  |  | Bauza-Ribot et al. 2012 |
| KC315638 | *Niphargus foreli* | n/a | n/a |  |  | McInerney et al. 2014 |
| HQ535705 | Chiltoniidae sp. | n/a | n/a |  |  | Guzik et al. 2011 |
| KJ661100 | *Austrochiltonia subtenuis* | n/a | n/a |  |  | King and Leys 2014 |
| KC578473 | *Platorchestia pacifica* | n/a | n/a |  |  | Yang et al. 2013 |
| KT209402 | *Gammarellus homari* | n/a | n/a |  |  | Raupach et al. 2015 |
| KT209051 | *Ericthonius punctatus* | n/a | n/a |  |  | Raupach et al. 2015 |
| HM053994 | *Abyssorchomene plebs* | n/a | n/a |  |  | Havermans et al. 2011 |
| JQ423242 | *Pseudorchomene debroyeri* | n/a | n/a |  |  | d'Udekem et al. (2012) |
